# Radiation Exposure and Frequency of Dental, Bitewing and Occlusal Radiographs in Children and Adolescents

**DOI:** 10.3390/jpm13040692

**Published:** 2023-04-20

**Authors:** Ina Manuela Schüler, Christoph-Ludwig Hennig, Rika Buschek, Rebecca Scherbaum, Collin Jacobs, Marcel Scheithauer, Hans-Joachim Mentzel

**Affiliations:** 1 Section Preventive Dentistry and Pediatric Dentistry, Department of Orthodontics, Jena University Hospital, 07743 Jena, Germany; 2 Department of Orthodontics, Jena University Hospital, 07743 Jena, Germany; 3 Section of Pediatric Radiology, Department of Radiology, Jena University Hospital, 07747 Jena, Germany; 4 Radiation Protection, Centre for Health and Safety Management, Jena University Hospital, 07747 Jena, Germany

**Keywords:** dental X-rays, dental radiology, effective dose, X-ray in children, radiograph, radiation exposure, single tooth radiographs, bitewing

## Abstract

Dental radiographs are valuable diagnostic aids for oral healthcare, but exposure to ionizing radiation carries health risks, especially in children due to their high radio-sensitivity. Valid reference values for intraoral radiographs in children and adolescents are still missing. This study aimed to investigate the radiation dose values and underlying justifications of dental, bitewing and occlusal X-rays in children and adolescents. Data from routinely executed intraoral radiographs between 2002 and 2020 with conventional and digital tube-heads were extracted from the Radiology Information System. The effective exposure was calculated from technical parameters and statistical tests performed. A total number of 4455 intraoral (3128 dental, 903 bitewing and 424 occlusal) radiographs were investigated. For dental and bitewing radiographs, the dose area product (DAP) was 2.57 cGy × cm^2^ and the effective dose (ED) 0.77 µSv. For occlusal radiographs, the DAP was 7.43 cGy × cm^2^ and the ED 2.22 µSv. Overall, 70.2% of all intraoral radiographs were dental, 20.3% bitewing and 9.5% occlusal radiographs. The most frequent indication for intraoral radiographs was trauma (28.7%), followed by caries (22.7%) and apical diagnostics (22.7%). Moreover, 59.7% of all intraoral radiographs were taken in boys, especially for trauma (66.5%) and endodontics (67.2%) (*p* ≤ 0.00). Girls were significantly more frequently X-rayed for caries diagnostics than boys (28.1% vs. 19.1%, *p* ≤ 0.00). The average ED of 0.77 µSv for intraoral dental and bitewing radiographs in this study was within the range of other reported values. The technical parameters of the X-ray devices were found at the lowest recommended levels to best limit the radiation exposure and to assure acceptable diagnostic efficacy. Intraoral radiographs were performed predominantly for trauma, caries and apical diagnostics—reflecting general recommendations for the use of X-rays in children. For improved quality assurance and radiation protection, further studies are necessary to determine the meaningful dose reference level (DRL) for children.

## 1. Introduction

Dental radiographs are valuable diagnostic aids for oral healthcare in children and adolescents. They add important findings to the clinical examination and help to more comprehensively diagnose and monitor oral diseases and evaluate traumata or tooth development and pathologies. Intraoral radiographs, one of the most common procedures in dentistry, are executed with the image detector placed intraorally and the radiation source extraorally. They comprise dental (or periapical), bitewing and occlusal radiographs.

In Germany, dental X-ray procedures account for 39% of all X-ray examinations but contribute only 0.3% to the total collective effective dose [1]. These national values are in line with the European values published by the European Commission [2]. Even so, efforts should be undertaken to keep the exposure to ionizing radiation as low as diagnostically achievable, especially in children and adolescents, who will undergo many examinations with ionizing radiation in the course of their lives [3].

Generally, the health risks associated with X-ray diagnostics are due to deterministic or stochastic radiation effects. Deterministic effects require a high radiation dose and result from killing cells. Conventional dental imaging never causes deterministic effects [4]. Stochastic effects result from damaging DNA. These effects might appear after very low exposure and there is no evidence of a threshold dose [4]. Furthermore, these risks might extend over the lifetime of an individual [5]. Children are particularly vulnerable to radiation damage due to the higher cell division rate, the higher proportion of water in children’s tissues, the close proximity of radiation-sensitive organs (e.g., lens, pituitary and thyroid glands and oral cavity) and the longer expected lifetime after exposure. Furthermore, specific biomarkers indicating genotoxic effects increased significantly in the oral mucosa of children several days after bitewing or panoramic radiographs [6]. Although the effective doses resulting from intraoral dental radiographs are low, tissues and organs are exposed and potentially endangered. In the literature, there are estimates of increased risks of cancer development due to the stochastic accumulation of low-dose radiation according to the no-threshold hypothesis. Comparing the estimated Life Attributable Risk of cancer incidence, female children seem to be at higher risk than males, and Cone Beam Computed Tomography scans represent an even higher risk regardless of gender [7,8]. Therefore, every effort should be taken to reduce radiation exposure in children and adolescents. Regarding stochastic exposure, risk estimation is difficult, but exposure limits for individuals in the low-dose range, including diagnostic radiology, are much needed [4].

Recently, pediatric dentistry associations worldwide updated their recommendations for the use of radiographs in children. Due to the high prevalence and burden of dental caries in childhood in the last few decades, X-ray screening depending on the individual caries risk was recommended in the past [9]. Today, X-ray screenings are no longer recommended. The policy document of the European Association of Paediatric Dentistry (EAPD) for prescribing dental radiographs in children and adolescents recommends an individualized and patient-specific justification for X-ray diagnostics as best clinical practice [3]. Likewise, the Guidelines of the American Academy of Pediatric Dentistry (AAPD) for prescribing dental radiographs for infants, children, adolescents and individuals with special healthcare needs emphasize that the timing of the radiographic examination should not be based upon the patient’s age, but upon each child´s individual circumstances [10]. It is also noted that every effort must be made to minimize the patient´s exposure to radiation. The As Low As Reasonable Achievable (ALARA) principle has been modified to As Low As Diagnostically Acceptable (ALADA) [11], reflecting the trade-off between image quality and radiation dose. The intention is to use the lowest radiation dose needed to reach sufficient image quality for the specific diagnostic task in the specific patient. For this concept, the term As Low As Diagnostically Achievable Being Indication-Oriented and Patient-Specific (ALADAIP) was introduced [12].

In the literature, there are heterogenic reports on the assessed effective dose of different dental examination modalities [4,13,14] and no authoritative statements on recommended protocols regarding radiation exposure among children and adolescents in dentistry.

The aim of this study was to investigate the number and dose values of dental, bitewing and occlusal X-rays in children and adolescents aged up to 18 years attending the Jena University Hospital for outpatient dental care between 01/2002 and 07/2020. The investigation of intraoral radiographs focused on assessing the dose area product of a single exposure and the resulting effective dose. Additionally, indications for intraoral X-rays were analyzed as age- and gender-related.

## 2. Materials and Methods

Data from routinely executed intraoral radiographs between 1 January 2002 and 31 July 2020 in patients aged up to 18 years were extracted from the Radiology Information System (RIS) of i-SOLUTIONS Health GmbH (RadCentre, Mannheim, Germany) and the Picture Archiving and Communication System (PACS) of GE Healthcare GmbH. All available intraoral radiographs were included, without any exclusion criteria. The University Hospital Computer Centre performed the data extraction from the databases.

The following X-ray-related parameters were collected: hospital information system identification number, patient´s date of birth and gender, date of X-ray execution, dose area product, indication and modality of X-ray. The patient´s date of birth and gender was used to build a pseudonym. There were several incomplete data sets in the extracted database. If possible, missing information was completed by manually accessing the RIS and transferring respective parameters into the database. Modalities comprised dental, bitewing and occlusal radiographs. There was no distinction made between right and left bitewing or maxillary and mandibular occlusal radiographs. Indications for the investigated intraoral radiographs were grouped into nine categories: caries diagnostics; apical diagnostics; endodontics; focus detection; trauma; detection of developing teeth, orthodontics; dental surgery; and prosthodontics.

During the study period, several dental X-ray machines served for radiologic diagnostics. Until May 2010, the tube-heads Heliodent (Dentsply Sirona, Bensheim, Germany) and ORALIX 65S (Gendex Corp., Lake Zurich, IL, USA) were used with conventional plain films. From November 2004, the first digital X-ray machine, Heliodent DS (Dentsply Sirona, Bensheim Germany), and later, from October 2012, the digital Heliodent Plus (Dentsply Sirona, Bensheim Germany), started their service.

The dose area product (DAP), a parameter in dosimetry for determining the amount of energy from ionizing radiation and the radiation exposure, was captured for each of the modalities. The DAP records the incident dose over the irradiated area (Gy × cm^2^). The DAP is proportional to the field size, if other parameters such as the tube voltage (kV), current time product (mAs) and focus–object distance remain the same. The effective dose (ED) is a measure of the biological effect of the ionizing radiation. The calculation of the ED requires a conversion factor (CF). According to Gosch et al. and Looe et al., the CF for skull examinations of 0.03 mSv/(Gy × cm^2^) was defined [14,15]. This decision was based on the technical settings of the X-ray machines: 60 kV tube voltage, 7 mA current time product and 0.1 sec exposure time. The following formula served to calculate the ED:

ED [mSv] = DAP [cGy × cm^2^] × CF [mSv/Gy × cm^2^]

Statistical data analysis was carried out with IBM SPSS Statistics 27. Descriptive statistics were performed using absolute and relative frequency distributions for categorical data, such as year, age, indication and gender. Mixed models with patient age as a dependent variable were calculated. A random effects model was modulated with patient age as an outcome variable and the patient as a random effect. Pairwise comparisons and cross-tabulations served to compare the different modalities with each category. All multiple tests were corrected via Bonferroni. The Fischer test served to analyze statistical differences, indications and X-ray modalities between gender groups. Statistical significance was set at *p* ≤ 0.05.

## 3. Results

**Figure 1:**
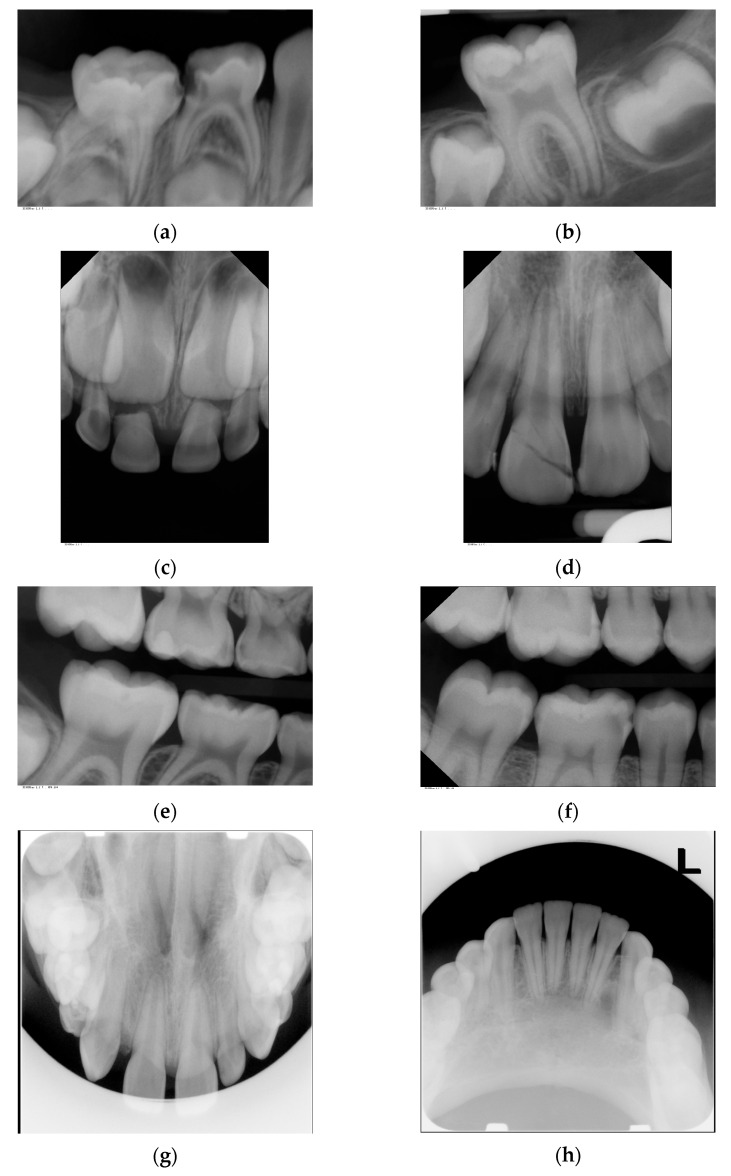
Examples of investigated intraoral radiographs: (**a**) dental radiograph of primary molars for caries and apical diagnostics; (**b**) dental radiograph of permanent molar for caries progression and apical diagnostics; (**c**) dental radiograph of primary teeth for trauma diagnostics; (**d**) dental radiograph of permanent teeth for trauma diagnostics; (**e**) bitewing radiograph for caries detection in primary molars; (**f**) bitewing radiographs for caries diagnostics in permanent molars; (**g**) occlusal radiograph for diagnosis of tooth germ position in the upper jaw; (**h**) occlusal radiograph for diagnosis of pathology in the lower jaw.

The present evaluation was based on a total number of 4455 intraoral radiographs taken in 2195 patients. In total, 3128 dental, 903 bitewing and 424 occlusal X-rays were included. Examples of the intraoral X-ray images are shown in Figure 1.

### 3.1. Dose Values

The dose area product and the effective dose for dental, bitewing and occlusal radiographs executed with the X-ray machines at Jena University Hospital are presented in Table 1.

Due to the greater exposed area and the different tissues under exposure, occlusal radiographs produced a threefold higher effective dose compared to dental and bitewing radiographs.

### 3.2. Annual Frequencies of Intraoral Dental Radiographs in Children and Adolescents

**Figure 2:**
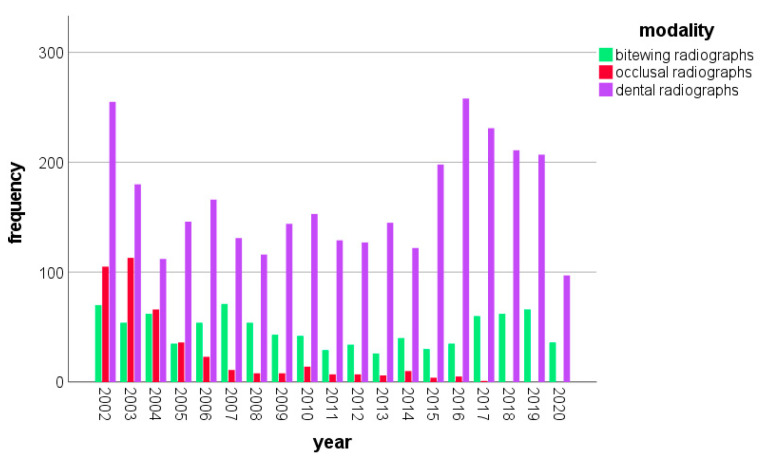
Number of intraoral dental, bitewing and occlusal radiographs per year in children and adolescents aged up to 18 years between 2002 and 2020 (in the year 2020, between 1 January and 31 July).

From 1 January 2002 to 31 July 2020, a total number of 4455 intraoral radiographs were taken in 2195 children and adolescents aged up to 18 years at Jena University Hospital. Variations regarding the frequency of X-ray diagnostics are observable—in terms of overall frequency and in terms of modality frequencies (Figure 2). In the year 2002, the total number of intraoral radiographs reached the highest value within the study period (*n* = 430), whereas the lowest number was registered in 2011 (*n* = 165). The annual frequency of intraoral X-rays decreased from 2002 to 2011, but increased again from 2015 to 2019 (Figure 2). Please note that data for the year 2020 were collected only until July.

Dental radiographs were the most frequently executed modality in all years. In particular, 70.2% (*n* = 3.128) of all intraoral radiographs were dental radiographs, followed by 20.3% (*n* = 903) bitewing radiographs. The least frequent modality was occlusal radiographs, amounting to 9.5% (*n* = 424). The number of occlusal radiographs decreased over the years, from *n* = 105 in 2002 and *n* = 113 in 2003 to zero in the years 2018, 2019 and 2020 (Figure 2). There was a change in the use of bitewing radiographs. Whereas, in 2002, bitewings accounted for 16.3% (*n* = 70) of all annual intraoral radiographs (*n* = 430), in 2019, the percentage was 24.2% (*n* = 66) of all 273 intraoral exposures.

While girls were X-rayed significantly more frequently than boys with bitewing radiographs (24.3% vs. 17.3%, *p* ≤ 0.00), intraoral dental radiographs were taken significantly more frequently in boys compared to girls (73.2% vs. 66.1%, *p* ≤ 0.00). Occlusal radiographs were executed in the same amount in both gender groups (9.4% in boys vs. 9.6% in girls, *p* = 0.88).

### 3.3. Indications for Intraoral Dental Radiographs

**Figure 3:**
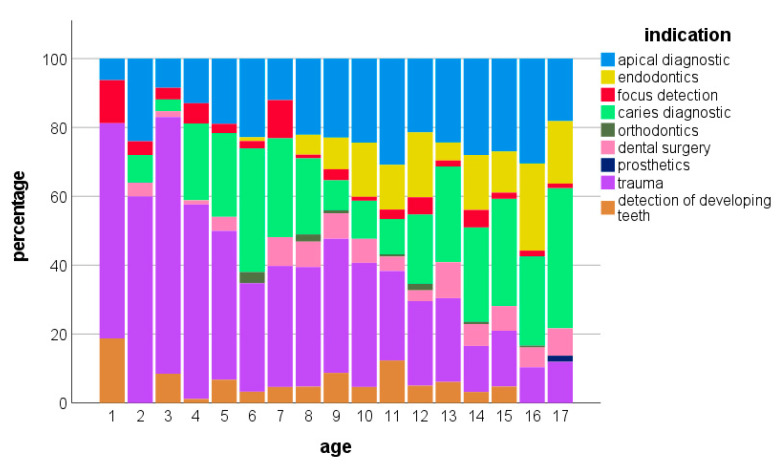
Age-related distribution of indications for intraoral radiographs.

The most frequent indication for intraoral radiographs was dental trauma (28.7%), followed by caries diagnostics (22.7%) and apical diagnostics (22.7%), while prostodontic or orthodontic indications remained below one percent (Table 2 and Figure 3). Table 2 displays the absolute and relative frequencies of indications for intraoral radiographs.

Over the years, most indications showed variations in their frequencies. Related to the total number of annual intraoral radiographs, those taken for apical diagnostics decreased from 29.8% in 2012 to 17.3% in 2020. Radiographs for caries diagnostics increased from 15.5% in 2015 to 36.8% 2020, and those executed for trauma diagnostics increased from 22.0% in 2012 to 37.7% in 2018. Certain indications, namely focus detection, orthodontics, prosthodontics and dental surgery, remained within the study period at a low level, with minor variations.

Further variations in indications for intraoral radiographs were observed in relation to the age of the patients. Children aged under four years underwent radiographs predominantly for trauma, followed by apical diagnostics and the detection of foci (Figure 3). Between the ages of four and eight years, besides trauma diagnostics, a remarkable share of radiographs for caries diagnostics occurred. A second increase in radiographs for caries diagnostics appeared from the age of twelve years, parallel with a decrease in trauma indications. Starting at eight years of age, an increasing number of radiographs accompanying endodontic treatments could be observed (Figure 3).

The youngest children receiving intraoral radiographs were 1.2 years old. Dental radiographs were executed on children from 1.2 years to 18 years of age (mean 11.7 years, SD: 4.3 years), bitewings from 4.2 years to 16 years of age (mean 13.5 years; SD: 3.8 years) and occlusal radiographs from 1.5 years to 18 years of age (mean 10.9 years; SD: 4.6 years). The mean age of girls was very slightly higher than the mean age of boys (12.2 years, SD: 4.3 years vs. 11.9 years, SD: 4.4 years).

Among the indications, patients X-rayed for dental trauma were the youngest (mean age: 9.4 years, SD: 4.0 years). The mean age in patients receiving radiographs for caries diagnostics was 12.4 years (SD: 4.3 years).

Comparing the patients by gender, boys were X-rayed more frequently than girls, and 59.7% of all intraoral radiographs were taken in boys. Especially for trauma (66.5%) and endodontics (67.2%), boys received significantly more radiographs than girls (33.5% for trauma and 32.8% for endodontics) (*p* ≤ 0.00). Girls were significantly more frequently X-rayed for caries diagnostics than boys (28.1% vs. 19.1%, *p* ≤ 0.00).

**Figure 4:**
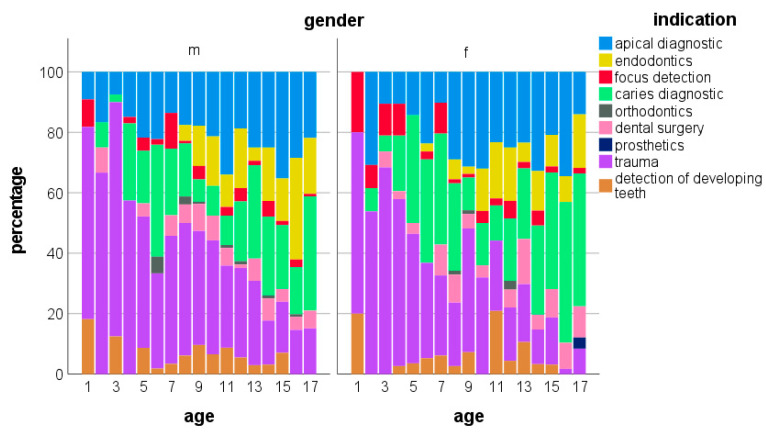
Age-related gender distribution of indications for intraoral radiographs.

In the age-related gender distribution of the indications for intraoral radiographs, small differences were noticeable. At an age under four years, more indications for trauma were noted in boys than girls (Figure 4). Similarly, at ages over six years, trauma indications dominated in boys. Beginning with the age of six years, girls were X-rayed more frequently for caries diagnostics compared to boys of the same age.

## 4. Discussion

Intraoral dental radiographs in children and adolescents are some of the most common procedures in dentistry and a valuable adjunct to the dentist’s clinical judgement for the diagnostics and monitoring of oral diseases in order to plan and provide the best dental treatment [10,16]. Dentists have to justify every radiograph for every patient individually, weighting the potential benefit (information to change treatments) against the potential radiation risk [12]. The present investigation contributes data on radiation exposure due to intraoral dental radiographs taken between 2002 and 2020 in children and adolescents.

The average effective dose of 0.77 µSv for intraoral dental and bitewing radiographs in this study was within the range of other reported values. Granlund et al. found an average effective dose of 0.8 µSv within a range of 0.1 to 2.6 µSv for intraoral dental radiographs and 0.3–1.4 µSv for bitewing radiographs, calculated as the sum of the phantom-based equivalent dose delivered to organs specified in ICRP 103, multiplied by their given tissue-weighting factors [17,18]. A recent review on doses for dental images summarized the effective doses for intraoral dental radiographs from the literature published between 2010 and 2020 and reported a higher mean effective dose of 1.32 µSv compared to the present study and a range between 0.60 and 2.56 µSv [13]. In both studies, the effective dose in panoramic radiographs was several times higher (36 µSv [17] and 17.93 µSv [13]). A retrospective cohort study investigating 4220 intraoral radiographs taken in patients up to 22 years of age between 2014 and 2015 observed an effective dose of 3.3 µSv, with the highest values in the age group of 0–6 years (7.6 µSv) [7]. These differences in effective doses might be explained by the variety of technical equipment and manufacturers, differences in technical settings and the uncertainty of the measurement methodology.

The measurement of the absorbed radiation dose can be attained by various dosimeters embedded in phantoms especially fabricated for radiation dosimetry in vitro. This method is used to calculate the effective dose of radiographic procedures [19] or absorbed organ doses [14,17,20] from the dosimeter readings under standardized but simulated conditions [21]. Another way to estimate the absorbed and effective dose is to apply a computer model. The Monte Carlo program PCXMC computes conversion coefficients from the dose area product [15,21], considering the tube voltage, filtration, projections, radiation field size and position. In the present study, published conversion coefficients were considered [14,15] and the most appropriate factor of 0.03 mSv/(Gy × cm^2^) chosen.

Effective dose calculation and reduction contributes to the protection of tissues and organs known to be vulnerable to ionizing radiation. Over the last few decades, along with technical enhancements in radiologic procedures, a continuing reduction in the adsorbed dose in the brain, lens of the eye and thyroid and salivary glands has been observed [22]. The most recent review of doses for dental imaging reported organ doses derived from intraoral dental radiographs: 0.63 µGy for bone marrow, 2.36 µGy for the brain, 22.79 µGy for salivary glands and 7.97 µGy for the thyroid gland [13]. The switch from film-based to digital X-ray machines led to a drastic improvement, lowering the effective dose and, consequently, the absorbed organ doses [19]. Nevertheless, although at a low level, intraoral dental radiographs produce radiation doses, especially in the tissues and organs located in the vicinity of the teeth. Dental and bitewing radiographs produce absorbed organ doses of up to approximately 20 µGy in the red bone marrow and salivary glands. Values of approximately 70 µGy for the brain and 50 µGy for the salivary glands have been detected for occlusal radiographs of the upper jaw [14]. A full-mouth bitewing examination produces more than 100 µGy in the salivary glands and more than 150 µGy in the oral mucosa [17]. The oral mucosa has moved to the focus of observation at the cellular level. The Buccal Micronucleus Cytome assay, a biomarker able to detect genetic damage, was utilized for buccal mucosal cells from children several days after radiographs were taken [6]. Bitewing radiographs produced a threefold increase in micronuclei, while digital panoramic radiographs produced a twofold increase in micronuclei [6]. With the understanding that intraoral dental radiographs are not harmless, it is of particular importance to establish diagnostic reference levels (DRL) for use in pediatric dentistry. Following the ICRP130 protocol, Looe et al. proposed E = 0.2–4.3 µSv as the DRL-associated effective dose for intraoral dental radiographs in children [14].

According to the technical parameters used for intraoral dental radiographs, the kilovoltage (kV) of the X-ray machines used in this study was 60 kV, the lowest value recommended for intraoral radiographs, which was reported according to a limited radiation dose and acceptable diagnostic efficacy [21,23]. Furthermore, the exposure time of 0.1 s was shorter compared to other studies [17], and small sensors of 2.2 × 3.5 cm were used for radiographs in children. Further potential to reduce the radiation exposure relies on the use of rectangular collimation [21].

In addition to uniform dose reference values, intervals between repeated exposures are also relevant for guidelines, so the extracted data were also evaluated with regard to justifying indications, X-ray modalities and the frequency distributions by age and gender. The aim was to identify periods in child development with the need for more frequent dental X-rays.

The increase in the overall number of radiographs in children and adolescents from 2015 onwards can be explained by structural and personal changes in the Department of Pediatric Dentistry at Jena University Hospital. The department was able to admit a higher number of patients due to an increase in medical and nursing staff.

In this investigation, the most frequent justification for intraoral radiographs was dental trauma. The use of radiographs in the diagnosis of dental trauma in children and adolescents supplements clinical examination and serves as a baseline to detect further sequelae [24,25]. Not all signs of injury will be evident clinically and radiographs enable a comprehensive diagnosis. Intraoral radiography better identifies traumatic dental injuries to primary teeth than a clinical examination alone [26]. Radiological detection of root fractures, alveolar bone fractures, the position of permanent tooth germs or periodontal pathologies has an important influence on the appropriate treatment decisions. According to the EAPD policy document regarding the best clinical practice for prescribing dental radiographs, dental (periapical) radiographs are the first choice for the radiological examination of dental trauma [3]. Furthermore, recent guidelines for managing dental trauma published by the American Academy of Pediatric Dentistry recommend the type and interval of follow-up radiographs in dental traumatology in both dentitions [27,28].

The young age of children X-rayed for trauma and the age-related pattern is consistent with other findings [29]. Often, a traumatic injury in primary dentition is the reason for the infant´s first visit to the dentist. The age distribution reflects a peak around age 2–3 years, which is the time at which children enjoy more mobility, leading to a higher risk of falls [29]. The higher number of indications for trauma seen at an age of under 4 years in boys than in girls coincides with other reports [29,30].

In addition to dental radiographs, intraoral bitewing radiographs were the second most frequent intraoral imaging procedure, accounting for 20.3% of all radiographs. Bitewing radiographs served predominantly for the detection and diagnosis of proximal caries in both dentitions. The mean age of children receiving bitewing radiographs (13.5 ± 3.8 years) was slightly higher that of children X-rayed for caries diagnostics (12.4 ± 4.3 years). In younger ages, dental radiographs were preferred in several cases for caries diagnostics due to the smaller sensor and the fact that there is no need for the child to close their mouth to hold the sensor.

In the literature, there is comprehensive evidence that without the use of dental radiographs, the caries prevalence would be underestimated in children and adolescents, mainly for caries lesions at proximal surfaces in molars [16]. Visual clinical examination accompanied by intraoral dental or bitewing radiographs detected up to 50% more proximal caries lesions in the posterior primary and permanent teeth compared to visual examination alone [16,31,32]. In children and adolescents, the value of detecting caries at an early stage lies in the possibility to start or intensify non-operative preventive measures, restore clinically sound dentin lesions before they progress and plan recall intervals [16]. However, it is recommended to consider dental radiographs as part of the initial dental examination of children but not to precede the clinical examination [16]. Furthermore, former recommendations of routinely repeated radiographs for caries detection screening were revised [3], focusing on more patient-specific, indication-oriented decisions. Radiographs provide information that is expected to contribute to better decisions regarding treatment options. Pontes et al. questioned the benefit of radiographs as a protocol in the diagnostic process to detect caries in children [33,34]. They observed only modest changes in the treatment decisions regarding primary molars made after additional radiographic evaluation compared to exclusively visual examination. For detecting caries, X-ray-free photo-optical alternative methods might be considered to enhance the accuracy of visual caries diagnostics and to support the treatment decision. Examples are near-infrared transillumination, laser fluorescence, fiber-optical transillumination or other fluorescence-based cameras [3].

Besides using alternative X-ray-free diagnostic devices, there are several possibilities to reduce radiation exposure in children and adolescents. It seems realistic to reduce the number of radiographs taken by avoiding screening or routine execution. Instead, strict and individualized justification should determine the prescription of each radiograph. A justified radiograph should make a substantial contribution to distinguishing between treatment options [3]. Optimization of the X-ray examination according to the ALADAIP concept requires optimal X-ray machine settings (exposure factor selection, beam size, field of view and image receptor). For intraoral radiography, protective devices such as rectangular collimation, fast image receptor speeds and thyroid shielding are able to reduce radiation exposure [3]. There is a certain health risk associated with every X-ray. It is our duty to keep this risk as low as possible. However, no X-ray should be withheld from the patient that is necessary for appropriate medical or dental care.

An important tool in quality assurance and radiation protection is the use of dose reference levels (DRL). For the X-ray examinations presented in this study, no corresponding DRL exist for children and adolescents. Further studies with larger sample sizes are necessary in order to determine the meaningful DRL for children.

A limitation of this study was the retrospective nature. Therefore, there was no individual analysis of comprehensive radiation exposure possible. Furthermore, some data were supplemented manually because not all data were stored in the digital system. The indications for radiographs were not recorded in the Radiology Information System until 2011. Furthermore, it was not possible to demonstrate a reduction in the effective dose due to the change from conventional film-based technology to digital X-ray technology, as the introduction of digital X-ray equipment was overlapping and took place during a transitional period, and the analogue images were digitized afterwards.

## 5. Conclusions

Intraoral radiographs were performed predominantly for dental trauma, caries diagnostics and apical diagnostics—reflecting general recommendations for the use of X-rays in children and adolescents. Regarding gender, boys were X-rayed more frequently than girls, especially for trauma and endodontics. Girls were significantly more frequently X-rayed for caries diagnostics than boys.

The average ED of 0.77 µSv for intraoral dental and bitewing radiographs in this study was low and within the range of other reported values. The technical parameters of the X-ray devices (kV and mAs) were found at the lowest recommended levels to best limit the radiation exposure and to assure acceptable diagnostic efficacy. Nevertheless, individualized justification for radiographs is mandatory to reduce radiation exposure.

For improved quality assurance and radiation protection, further studies are necessary to determine the meaningful dose reference level (DRL) for children.

## Figures and Tables

**Table 1:** Dose area product (DAP) and effective dose (ED) of dental, bitewing and occlusal radiographs executed with tube-head X-ray machines between 2002 and 2020 in patients aged up to 18 years.

Modality	DAP (CGy × cm^2^)	ED (µSv)
Dental radiograph	2.57	0.77
Bitewing radiograph	2.57	0.77
Occlusal radiograph	7.43	2.22

**Table 2:** Frequency of indications for intraoral radiographs.

Indication	Number of Radiographs	Percentage (%)
Apical diagnostics	495	22.7
Endodontics	244	11.2
Focus detection	68	3.1
Caries diagnostics	496	22.7
Orthodontic	15	0.7
Dental surgery	130	6.0
Prosthetics	4	0.2
Trauma	627	28.7
Detection of developing teeth	104	4.8
TOTAL	2138	100.0

## Data Availability

Raw data can be made available upon reasonable request.
